# Twitter reveals spatio-temporal variation in vaccine concerns in Sub-Saharan Africa

**DOI:** 10.1101/2025.08.19.25334033

**Published:** 2025-08-24

**Authors:** Devansh Jain, Sunny Rai, Juhi Mittal, Anietie Andy, Alison M. Buttenheim, Sharath Chandra Guntuku

**Affiliations:** 1 Language Technologies Institute, School of Computer Science, Carnegie Mellon University; 2 Department of Computer and Information Science, University of Pennsylvania; 3 Leonard Davis Institute of Health Economics, University of Pennsylvania; 4 Electrical Engineering and Computer Science Department, Howard University; 5 Department of Family and Community Health, University of Pennsylvania School of Nursing

## Abstract

**Background:**

COVID-19 vaccine hesitancy, fueled by concerns about vaccine development, side effects, and misinformation on social media platforms like Twitter, resulted in lower vaccination rates in Sub-Saharan Africa.

**Methods:**

We collected, preprocessed, and geolocated 6,546,893 tweets related to COVID-19 vaccination from Sub-Saharan Africa. Using a vaccine misinformation classifier trained on RoBERTa embeddings, we identified 371,965 tweets in our dataset that included misinformation. We characterized the relationship between specific COVID-19 vaccine topics and the prevalence of misinformation, examined temporal variation in misinformation, and separately described the prevalence of misinformation in clusters defined by country-level socioeconomic and development metrics and by COVID-19 epidemiology.

**Results:**

Misinformation in Sub-Saharan Africa is associated with discussions about pharmaceutical company profits, global access to vaccines and disparity, and trust in scientific research regarding vaccines. The prevalence of misinformation topics varied widely across country clusters as defined by socioeconomic development and COVID-19 epidemiology metrics.

**Conclusions:**

Social media data provides valuable insights about vaccine hesitancy and vaccine misinformation in Sub-Saharan Africa that can inform policy and programmatic interventions to support vaccine demand and vaccine promotion.

## 1. Introduction

On May 5, 2023, the World Health Organisation declared an end to COVID-19 as a global health emergency. The unprecedented global effort undertaken to develop and adopt highly effective vaccines against COVID-19 played a crucial role in the slowdown of the pandemic (1). However, while 171 vaccine doses per 100 population had been administered globally by May 2023, only 53 doses had been administered per 100 population in Africa (2). Two primary reasons for this are global vaccine distribution inequity (3) and vaccine hesitancy (4).

COVID-19 vaccine hesitancy has been driven primarily by concerns about the rapid development of vaccines, fear of side effects, and misinformation about vaccines on social media platforms (5). While social media platforms such as X (formerly Twitter) disseminate news, viewpoints, knowledge, and public health guidance around COVID-19 and vaccine-related issues, misinformation and intentional disinformation about COVID-19 and vaccines also circulate (6,7). Prior research has examined the association between COVID-19 vaccine misinformation and vaccine hesitancy (8) that misinformation promulgated over social media may have contributed to the public’s waning confidence and vaccine reluctance (9,10). The WHO coined the term “infodemic” to describe the rapid spread of COVID-19 and vaccine misinformation on social media platforms (11). Infodemic refers to “too much information including false or misleading information in digital and physical environments during a disease outbreak” (3).

X^1^ has been leveraged to measure changes in mental health (12), identify misinformation (13), study psychosocial effects (14), and uncover emerging symptoms (15). According to a review of 45 studies about COVID-19 vaccine misinformation on social media platforms (16), X (17–19) was the most studied platform. Social media provides rich, in-the-moment self-disclosed information on the attitudes towards and concerns about vaccination across communities. Social media data can also complement large-scale surveys, which are often challenging to field quickly in resource-constrained contexts in multiple languages. However, the vast majority of research on social media and misinformation generally, and on X and COVID-19 vaccine misinformation specifically, has been conducted using data from the United States and Europe (20,21). Less studied is the prevalence and characterization of online misinformation among non-Western Democratized Educated Industrial Democratic (WEIRD) countries. In this study, we 1) collect a large-scale dataset of COVID-19 vaccination-related tweets from 44 Sub-Saharan African countries (SSA)^2^ between December 2020 and September 2022, 2) build a machine learning-based classifier to identify misinformation-related tweets, and 3) characterize the longitudinal and geospatial landscape of COVID-19 vaccine misinformation across countries in SSA clustered on socioeconomic development and on COVID-19 epidemiology. We identify statistically significant patterning of COVID-19 vaccine topics and misinformation prevalence across clusters.

## 2. Materials and Methods

### 2.1 Data Collection and Pre-processing

In December 2020, we launched a data puller using the Python package TwitterMySQL^3^ to collect tweets matching at least one of the following keywords following previous work (20): *vaccine, vaccination, moderna, pfizer, #antivax, #CashingInOnCovid, #MyBodyMyChoice, #Vax* via the official Twitter Application Programming Interface (API) available at the time. The data puller continuously collected tweets from using the Stream API until September 30, 2022 collecting 587,152,450 tweets in total.

#### Geolocation:

Mapping tweets to geographical locations around the world is non-trivial (22), since only a small fraction of tweets have geolocation coordinates that can be mapped directly. Thus, we follow (39) and instead rely on parsing the free-response self-report location field that accompanies a tweet. This field sometimes contains an administration level just by itself (e.g., “Johannesburg”, “Gauteng”, “South Africa”), or multiple administration levels together (e.g., “Johannesburg, Gauteng”, “Gauteng, South Africa”), or even non-identifiable phrases (e.g., “Who cares man”).

We start by tokenizing strings with “,” as the delimiter. This is based on the assumption that different administration levels in a location string will be separated by a comma so that the number of “,” in a location string represents the number of administration levels mentioned. Given the number of administration levels, we then attempt to match the location (more details in the supplementary information) starting from the highest administration level.

We also utilize substring lookup for location strings having more than 1 administration level. Specifically, if the entire location string does not have a valid entry in its corresponding mapping dictionaries, we remove the lowest administration level from the string and repeat the mapping process. This is helpful when the lowest administration level in a string (usually city) is not present in the mapping dictionaries but the string contains information about higher administration levels that can be used to successfully identify its location (with a loss of granularity). We did not consider the Democratic Republic of Congo for two reasons. First, it was particularly difficult to differentiate DRC and Congo in self-reported free-form user locations that were used to geolocate tweets. Second, we encountered multiple variations of DRC in the user locations that made it difficult to map them appropriately using our heuristic-based geolocation algorithm.

In a random selection of 100 tweets, we found that this process correctly mapped 95% tweets. Further, the algorithm reduced false positives and improved time and space efficiency of the mapping process over the baseline.

We then used the geolocation algorithm to map tweets to countries in Sub-Saharan Africa and manually checked self-reported and mapped locations for a sub-sample of tweets from each country geolocated dataset included 6,541,866 tweets from Sub-Saharan Africa (*full* version: *D*_*full*_); after cleaning and removing duplicate tweets, the *clean* version of the dataset (*D*_*clean*,_ 2,634,189 tweets; 368951 unique users) was used for estimating topics and misinformation classification (see Table 1 and 2).

### 2.2 Topic modeling and annotation

We created a set of topics using Latent Dirichlet Allocation (LDA; (23)), a probabilistic generative model that assumes tweets are generated by a combination of topics (which are latent variables) and that topics are a distribution of words. We first removed the top 100 most frequent words in the dataset, along with a manually curated list of stopwords. Using the DLATK library’s (24) interface for the MALLET implementation of LDA (25), we generated 50, 100, and 200 topics, with an alpha level of 5. Following (26), we quantitatively assess the quality of topics in each set by computing coherence scores using four measures of coherence (See Appendix) (27). We also reviewed the top words of topics within each topic set for a qualitative check of interpretability. Using both methods, we reached the consensus that the 200-topic model was optimal. Finally, we obtained the probability distribution for each tweet across all 200 topics.

For ease of interpretation, we used ChatGPT (with GPT3.5) (28), a large language model, to generate topic labels for each topic using their top 10 words. Specifically, we prompted ChatGPT with the following instructions: “The following are topics associated with COVID-19 vaccination on X. The top words for each topic are listed. In each row, please find the relationship between words and conclude a topic with one short phrase. One author (AB) manually evaluated the quality of the LLM-generated topic labels and revised them where necessary.

### 2.3 Cluster identification

We wanted to study trends of vaccine concerns across multiple countries in Sub-Saharan Africa. Instead of looking at individual countries, we clustered countries in our dataset to investigate the differential spread of misinformation. The cluster set was computed by performing hierarchical clustering on COVID-19 case rate (number of cases/population), COVID-19 death rate (number of deaths/population) (29), COVID-19 vaccine rate (number of vaccinations/population) (30), GDP per capita, Corruption Perceptions Index (CPI), Democracy Index (DI), and cellular phone subscription rate (number of cellular phone subscriptions/population) for the countries in consideration (31). Using a dendrogram, we selected 3 clusters. Table 2 shows the cluster assignment for each country. Table 3 contains the distributions of tweets, users, retweets, and the number of countries for each cluster.

### 2.4 Misinformation Classification

We used the ANTiVax dataset (32) to train a supervised vaccine misinformation classifier. The ANTiVax dataset contains a total of 15,073 English tweets, 5751 of which were misinformation and 9322 general vaccine-related tweets. It was manually annotated using common myths regarding vaccine misinformation from reliable sources, and validated by medical experts in public health. We employed RoBERTa (33), a pre-trained contextual word embedding model to generate numeric vector representations of the annotated tweets. We computed tweet embeddings by averaging the *roberta-base* model 10^th^ layer embedding for each word in the tweet (34).

Next, we treated RoBERTa embeddings-based numeric representations of tweets as the independent variable and misinformation labels as the dependent variable to train a Random Forest Classifier (35) for misinformation. We used the default parameters in the DLATK library (24). Finally, we applied the trained classifier to *D*_*clean*_ to compute the probability of each tweet containing misinformation. Tweets with a misinformation probability above 0.6 were labeled as misinformation. The cutoff value was empirically determined to optimize the tradeoff between precision and recall. We manually labeled randomly sampled 100 tweets from our dataset to estimate the quality of the trained classifier. We found that our classifier correctly labeled 80% of the tweets. Out of the 100 tweets, the classifier labeled 14 as misinformation, out of which 5 were incorrectly labeled (Precision: 0.77, Recall: 0.72, F1-score: 0.74).

### 2.5 Differential Language Analysis

We performed a differential language analysis to identify statistically significant correlations between the topics and different outcomes. We extracted the quarterly prevalence of topics across tweets aggregated to the second-highest administration level (aka, province/state) to assess trends over time. Topics were then used as input in a logistic regression model with dummy variables for each quarter as the outcomes for temporal analyses. For the country clusters, we aggregated the probability distribution of tweet-topics to the second-highest administration level and used them as input in a linear regression model with dummy variables for each of the cluster sets as the outcomes. For the misinformation outcome, we conducted a tweet-level analysis with topics as independent variables in a linear regression model to predict the probability of each tweet including misinformation. Based on conventional linguistic analysis, we used a *p*-value of < 0.05 to identify significant linguistic markers, and all *p*-values were corrected for the false discovery rate during multiple hypothesis testing using the Bonferroni correction.

## 3. Results

### 3.1 Country Clusters

**Figure 1:**
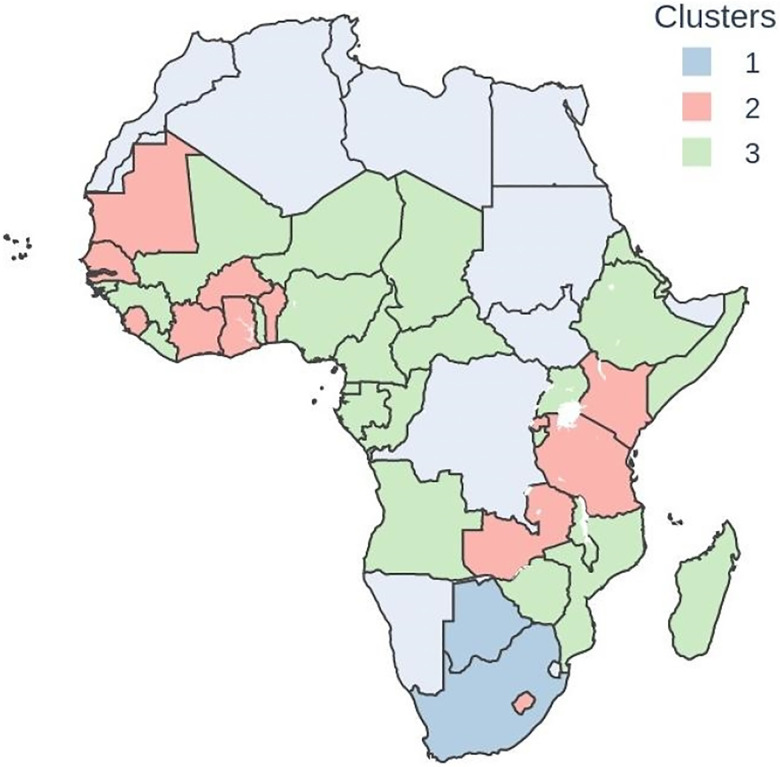
Choropleth map for clusters at the country level. Democratic Republic of Congo was excluded due to insufficient tweets (See Appendix for more details).

Three clusters emerged from the clustering analysis (see Table 3 for metrics used for clustering and Figure 1 for countries). Cluster 1 comprises 4 countries i.e., Botswana, Cape Verde, Mauritius, and South Africa; Cluster 2 has 14 countries such as Benin, Burkina Faso, Gambia, etc. and Cluster 3 has 25 countries such as Angola, Burundi, and Cameroon, etc.

### 3.2 Misinformation

Out of 2,636,397 tweets in *D*_*clean*_, our misinformation classifier predicted 371,965 tweets (14.11%) to be misinformation. The misinformation rate (number of misinformation posts/number of total posts) varied by cluster (see Table 2 for country-cluster mapping). Topics most strongly associated with misinformation included viral variants and mutations, profits of pharmaceutical companies, COVID-19 measures and restrictions, political and societal issues, African countries’ ability to produce vaccines, global access to vaccines and disparity, liability of vaccine manufacturers, global access to COVID-19 Vaccines, trust in medical professionals and scientific research regarding vaccines, and experimental treatments and drugs (see Table 4, Top pane). Topics least associated with misinformation included experiences with vaccine registration and appointment scheduling, dose administration and booster shots, lack of information or knowledge, Vaccine sites and rollout updates, Personal experiences with Vaccination, Instructions on how to register or find vaccine-related information, Age-specific vaccine registration and eligibility criteria, Vaccination sites and programs, Vaccination Centers and Appointments, and Polite requests for information or assistance (see Table 4, Bottom pane).

### 3.3 Topic Analysis by Cluster

**Figure 2:**
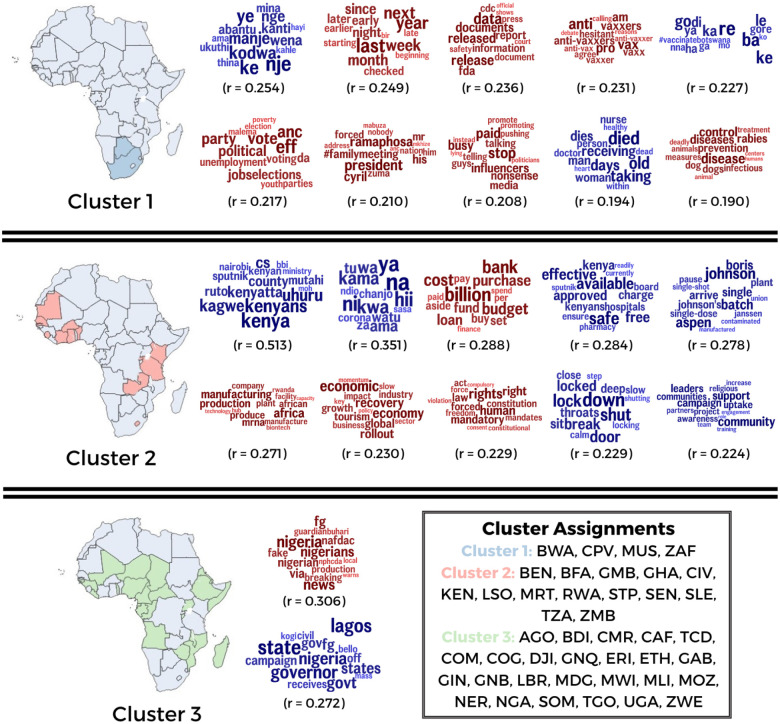
Topics associated with clusters. Word clouds for the top 10 topics associated with each cluster are shown here. Topics that are positively associated with misinformation are shown in red whereas the ones negatively associated with misinformation are shown in blue. Only Pearson *r* that are significant at *p* < 0.05 with Bonferroni correction are presented.

We also identified topics most associated with different clusters. Out of 200 topics, 58 varied significantly by cluster (see Figure 2). Topics prevalent in Cluster 1 included release of official information, vaccine hesitancy, criticism of influencers and media, and control and prevention of infectious diseases. Topics prevalent in Cluster 2 included the COVID-19 situation in Kenya, funding for vaccines, vaccine manufacturing in Africa, and economic impact of COVID-19. Topics prevalent in Cluster 3 included COVID-19 situation in Nigeria and COVID-19 response in Nigeria.

### 3.4 Topic Analysis Over Time

**Figure 3:**
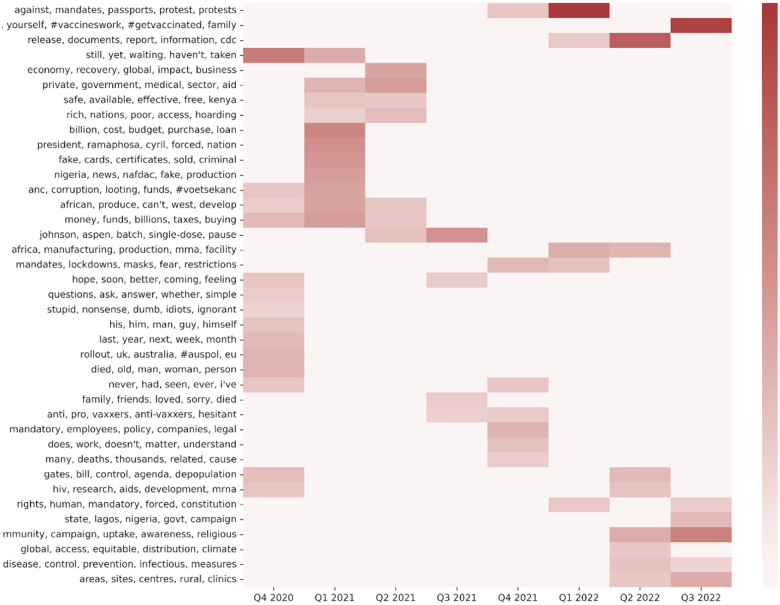
Topics significantly associated with clusters quarterwise.

Topic prevalence varied significantly by calendar quarter (see Figure 3). Among 58 topics significantly associated with country clusters, the topics most prevalent in the last quarter of 2020 included deaths, global vaccine rollout efforts, and funding for vaccines. Topics most prevalent in the first and second quarters of 2021 include private and government sector involvement, African countries’ ability to produce vaccines, and funding for vaccines. Topics most prevalent in the third quarter of 2021 included Johnson & Johnson’s vaccine batch issue and vaccine hesitancy. In the fourth quarter of 2021, vaccine hesitancy remained a prevalent topic, along with legal aspects of vaccine mandates in the workplace and COVID-19 measures and restrictions. Discussions about protests against vaccine mandates and vaccine manufacturing in Africa were prevalent in the first quarter of 2022, while prevalent themes in the second and third quarters of 2022 shifted to community initiatives to promote vaccine uptake, encouragement to get vaccinated, and information about vaccination centers.

## 4. Discussion

In this first-of-its-kind analysis of tweets originating in Sub-Saharan Africa regarding the COVID-vaccine, we identified significant variations in the prevalence of latent topics over time and across clusters of countries in the region defined by sociodemographic and epidemiologic characteristics. Temporal trends in topic prevalence mirror phases of the pandemic and reflect the news cycle as well, particularly around the development and roll-out of vaccines. Socio-spatial variation in topic prevalence reflected the different epidemiologic, sociopolitical, and economic contexts in which the pandemic and vaccine roll-out were experienced in the region. While about 1 in 7 tweets in the full dataset contained misinformation, this rate varied substantially over the socio-spatial clusters and over time.

Our analysis makes several unique contributions to our understanding of the role of misinformation in COVID-19 vaccine hesitancy in a region that ultimately had low population-level COVID-19 vaccine coverage (2) First, while most published studies on COVID-19 vaccine hesitancy in Sub-Saharan Africa rely on self-reported attitudes, beliefs, and information-seeking behaviors collected via survey (36–38), our analysis is based on natural language posts generated by social media users. Second, our unique clustering of countries by sociodemographic and epidemiologic characteristics reveals a more nuanced description of vaccine hesitancy and misinformation than single-country (22) or sub-regional (39) analyses can offer; these insights can enhance targeted and precise public communication. A third important contribution is the refinement and finetuning of the RoBERTa model to detect misinformation tweets originating specifically from SSA countries. This allows us to describe temporal trends in the progression of misinformation over a span of two years, from initial claims about global vaccine access to more specific topics about local vaccine availability and government programs.

A final important contribution is the identification within the misinformation-containing tweets of specific themes related to vaccine availability, government administration of vaccines, and COVID-19 treatment. This association also varied by country cluster. In the first cluster, (comprising South Africa, Botswana, Mauritius, and Cape Verde), prevalent topics within misinformation-containing tweets included state administration/elections, the influence of paid influencers/media, and concerns about infections/diseases transmitted by animals. The second cluster (including Zambia, Tanzania, and Kenya) featured topics such as the impact on the economy/tourism, reliance on the West for COVID-19 vaccines, and constitutional rights in the context of COVID-19 mandates and restrictions in the corpus of misinformation-containing tweets. In the third cluster (with countries such as Nigeria, Angola, Niger, and Chad), topics positively associated with misinformation centered on state approval and vaccine administration, specifically Nigeria’s NAFDAC (Food and Drug) and NPHCDA (Healthcare).

Our study does have some important limitations. Our misinformation detection model exhibits low precision, primarily attributed to the prevailing tone of fear in COVID-19-related tweets. We speculate that this might have led to a weak correlation between vaccination rate and misinformation rate. This fear-inducing tone is often found in tweets containing misinformation. Developing a more nuanced model capable of contextualizing fear (such as distinguishing fear related to vaccines from fear related to infection) would lead to more precise predictions. Additionally, there is an urgent need to enhance text analysis capabilities for African languages, allowing for a better representation of misinformation patterns associated with the specific cultural context of interest. However, given the paucity of published research analyzing geotagged social media posts from Sub-Saharan Africa, our study represents an important contribution.

It is highly likely that the influence of social media on the health information ecology in Sub-Saharan Africa will continue to grow (40). It is also almost certain that future epidemics and pandemics will require rapid scale-up and public delivery of novel vaccines. Understanding how misinformation spreads in this context and how topics and tone both reflect and can predict public sentiment and attention will continue to be vital in the design of public health promotion campaigns and efforts to counter misinformation. Advancing methods for social media analysis applied in WEIRD contexts for the LMIC context is crucial.

## Supplementary Material

1

## Figures and Tables

**Table 1:** Language-wise descriptive counts for two versions of the dataset.

*D*_*Full*_		*D*_*Clean*_	
Language	Posts	Language	Posts
English	5,996,367	English	2,383,738
Undetermined	185,501	French	85,243
French	143,916	Undetermined	36,832
Indonesian	41,760	Indonesian	26,371
Tagalog	40,145	Tagalog	24,070
Spanish	20,591	Spanish	13,371
Italian	15,409	Italian	8,676
Haitian	11,395	Haitian	7,126
German	10,553	Estonian	6,288
Estonian	9,390	German	5,822

**Table 2:** Country-wise descriptive counts and cluster assignments for the full and clean versions of the dataset. Countries are arranged alphabetically.

		*D*_*Full*_			*D*_*Clean*_		
Country	Cluster	Tweets	Users	Retweets	Tweets	Users	Retweets
Angola	3	7,621	1,174	5,039	4,067	714	1,919
Benin	2	4,903	601	3,899	1,977	386	1,074
Botswana	1	200,266	12,228	128,827	87,898	8,517	22,799
Burkina Faso	2	72,797	2,566	57,251	37,164	1,818	23,335
Burundi	3	3,832	646	2,565	2,093	469	918
Cameroon	3	88,070	9,290	57,978	38,943	6,262	11,820
Cape Verde	1	1,638	284	931	878	189	232
Central African Republic	3	23,252	1,316	16,846	8,845	909	2,974
Chad	3	2,998	314	1,522	1,764	231	366
Comoros	3	252	56	156	136	37	47
Congo	3	15,629	1,859	7,518	8,616	1,408	2,959
Djibouti	3	5,431	464	2,848	3,151	326	1,018
Equatorial Guinea	3	1,251	93	979	611	63	347
Eritrea	3	1,593	224	1,200	634	147	279
Ethiopia	3	39,940	5,675	29,806	13,713	3,049	6,152
Gabon	3	6,963	758	4,493	3,589	561	1,269
Gambia	2	4,732	743	3,294	2,464	506	1,134
Ghana	2	353,801	36,741	256,308	130,081	21,611	46,669
Guinea	3	3,296	323	2,550	2,009	224	1,333
Guinea-Bissau	3	52	23	33	21	14	4
Ivory Coast	2	18,230	2,379	13,639	8,506	1,434	4,394
Kenya	2	652,769	72,935	463,636	249,830	43,170	80,080
Lesotho	2	10,553	1,343	5,942	5,589	943	1,380
Liberia	3	147,515	7,083	93,939	63,512	5,126	14,563
Madagascar	3	18,153	1,246	13,489	8,344	898	4,013
Malawi	3	23,263	2,968	13,678	11,874	2,047	3,464
Mali	3	21,948	1,281	13,682	10,848	913	3,371
Mauritania	2	1,253	269	877	629	182	285
Mauritius	1	11,276	637	5,730	6,632	441	1,340
Mozambique	3	6,713	792	4,382	3,329	556	1,216
Niger	3	2,989	540	1,874	1,669	405	623
Nigeria	3	874,109	132,921	592,504	328,484	69,876	92,879
Rwanda	2	57,265	6,581	42,832	19,287	3,663	6,917
Sao Tome and Principe	2	18	1	10	7	1	0
Senegal	2	30,260	4,590	21,341	14,595	2,609	6,656
Sierra Leone	2	4,490	840	2,341	2,577	561	662
Somalia	3	21,958	2,563	15,390	9,947	1,683	3,991
South Africa	1	3,283,792	211,229	2,207,271	1,300,925	135,484	353,443
Tanzania	2	9,300	1,012	6,542	4,700	697	2,277
Togo	3	3,504	347	2,156	2,307	241	1,019
Uganda	3	309,750	34,080	216,546	131,596	23,040	46,073
Zambia	2	32,961	5,646	19,501	16,869	3,824	4,369
Zimbabwe	3	161,480	16,565	98,027	83,479	11,737	23,222
**Total**		**6,541,866**	**583,226**	**4,439,372**	**2,634,189**	**356,972**	**782,885**

**Table 3:** Descriptive statistics for country clusters and mean socioeconomic development and COVID-19 epidemiology metrics. COVID-19 metrics are reported per 100,000.

Cluster number: countries	1: Botswana, Cape Verde, Mauritius, South Africa	2: Benin, Burkina Faso, Gambia, Ghana, Ivory Coast, Kenya, Lesotho, Mauritania, Rwanda, Sao Tome and Principe, Senegal, Sierra Leone, Tanzania, Zambia	3: Angola, Burundi, Cameroon, Central African Republic, Chad, Comoros, Congo, Djibouti, Equatorial Guinea, Eritrea, Ethiopia, Gabon, Guinea, Guinea-Bissau, Liberia, Madagascar, Malawi, Mali, Mozambique, Niger, Nigeria, Somalia, Togo, Uganda, Zimbabwe
**#Tweets**	3,496,972	1,253,332	1,791,562
**#Users**	224,306	136,160	222,417
**#Retweets**	2,342,759	897,413	1,199,200
**#Countries**	4	14	25
**Case Rate (per 100K)**	12995.573	811.269	537.960
**Death Rate (per 100K)**	107.999	12.027	7.858
**Vaccine Rate (per 100K)**	134044.924	62774.579	38249.532
**GDP per capita**	6564.934	1483.938	1664.530
**Corruption Perceptions Index (CPI)**	53.250	38.714	25.120
**Democracy Index (DI)**	7.643	4.808	3.250
**Cellular Subscriptions per capita**	146.437	107.881	69.646

**Table 4:** Topics associated with misinformation. ChatGPT generated Topic Labels, Top words, Pearson *r*, and confidence intervals for the top 10 positively and negatively associated topics are shown here.

Positive Topics			
Topic Label	Top Words	r	CI
Viral variants and mutations	virus, new, variants, mutations, strains, spread	0.171	0.17, 0.172
Profits of pharmaceutical companies	companies, money, pharmaceutical, business, profit, billions	0.147	0.146, 0.148
COVID-19 Measures and Restrictions	mandates, lockdowns, passports, masks, fear, restrictions	0.135	0.134, 0.136
Political and societal issues	political, change, issue, society, power, opinion	0.132	0.131, 0.134
African countries’ ability to produce vaccines	countries, african, produce, can’t, west, develop	0.131	0.13, 0.132
Global Access to Vaccines and Disparity	rich, global, poor, access, hoarding, developing	0.130	0.128, 0.131
Liability of vaccine manufacturers	companies, manufacturers, liability, accountable, injury, compensation	0.124	0.123, 0.125
Global Access to COVID-19 Vaccines	waiver, global, access, patent, production, ip	0.121	0.12, 0.123
Trust in medical professionals and scientific research	medical, science, doctors, experts, research, trust	0.118	0.117, 0.119
Experimental treatments and drugs	treatment, ivermectin, drugs, experimental, cure, medicine	0.115	0.113, 0.116
Negative Topics			
Topic Label	Top Words	r	CI
Experiences with vaccine registration and appointment scheduling	registered, appointment, waiting, sms, site, book	−0.177	−0.178, −0.175
Dose administration and booster shots	dose, second, first, shot, booster, jab	−0.169	−0.17, −0.168
Lack of Information or Knowledge	know, where, say, didn’t, anyone, anything	−0.167	−0.168, −0.165
Vaccination sites and rollout updates	site, jab, drive, centre, #vaccinerolloutsa, #ichoosevaccination	−0.156	−0.157, −0.155
Personal Experiences with Vaccination	arm, side, pain, effects, shot, days	−0.127	−0.128, −0.126
Instructions on how to register or find vaccine-related information	register, visit, find, information, call, site	−0.123	−0.124, −0.122
Age-specific vaccine registration and eligibility criteria	years, over, age, 18, 60, 35	−0.110	−0.111, −0.109
Vaccination sites and programs	sites, #ichoosevaccination, gauteng, mec, #vaccinerolloutsa, drive	−0.108	−0.109, −0.107
Vaccination Centers and Appointments	centre, hospital, tomorrow, center, appointment, clinics	−0.105	−0.106, −0.103
Polite requests for information or assistance	please, sites, thanks, link, information, kindly	−0.103	−0.104, −0.102

## Footnotes

1 We used the term X and Twitter interchangeably.

2 Sub Saharan Africa countries - https://en.wikipedia.org/wiki/Sub-Saharan_Africa#Demographics

3
https://github.com/dlatk/TwitterMySQL
