# Supplementary material for: Twitter reveals spatio-temporal variation in vaccine concerns in Sub-Saharan Africa
Source: medRxiv. 2025 Aug 24:2025.08.19.25334033. Preprint. [Version 1] doi: 10.1101/2025.08.19.25334033 (PMC12393616; doi:10.1101/2025.08.19.25334033)
Supplement: 1 [file NIHPP2025.08.19.25334033V1-supplement-1.pdf]

medRxiv preprint doi: https://doi.org/10.1101/2025.08.19.25334033; this version posted August 24, 2025. The copyright holder for this preprint (which was not certified by peer review) is the author/funder, who has granted medRxiv a license to display the preprint in perpetuity.
It is made available under a CC-BY 4.0 International license .

Supplementary Information

A.1 Data Collection and Pre-processing

We used a geolocation algorithm (described in Sections A.2 and A.3) to map tweets to countries from Sub-Saharan Africa. Next, we randomly collected samples of up to 3000 tweets for each country from the mapped set and manually checked if the self-reported location and mapped location corresponded. This step helped eliminate false positives due to ambiguous self-reported locations. From the geolocated dataset, we retained only tweets originating from Sub-Saharan Africa. We also cleaned the data by mapping URLs and user mentions (@-mentions) to special tokens and removing duplicate tweets based on tweet id and tweet content. The language of tweet is automatically detected by the Twitter API (in ISO 639 language codes[5]). In compliance with Twitter's Terms of Service, we only include each tweet's numeric tweet id (status id; as given by the Twitter API) in the public release of the datasets.

A.2 Geolocation Algorithm

Mapping tweets to geographical locations around the world is non-trivial (38), since only a small fraction of tweets have geolocation coordinates that can be mapped directly. Thus, we follow (39) and instead rely on parsing the free-response self-report location field that accompanies a tweet. This field sometimes contains an administration level just by itself (e.g., "Johannesburg", "Gauteng", "South Africa"), or multiple administration levels together (e.g., "Johannesburg, Gauteng", "Gauteng, South Africa"), or even non-identifiable-phrases (e.g., "Who cares man").

We start by tokenizing strings with "," as the delimiter. This is based on the assumption that different administration levels in a location string will be separated by a comma so that the

[5] ISO 639-2 https://www.loc.gov/standards/iso639-2/php/code_list.php

medRxiv preprint doi: https://doi.org/10.1101/2025.08.19.25334033; this version posted August 24, 2025. The copyright holder for this preprint (which was not certified by peer review) is the author/funder, who has granted medRxiv a license to display the preprint in perpetuity.
It is made available under a CC-BY 4.0 International license .

number of “,” in a location string represents the number of administration levels mentioned. Given the number of administration levels, we then attempt to match the location string with the mapping dictionaries detailed in Section A.3, starting from the highest administration level.

We also utilize sub-string lookup for location strings having more than 1 administration level. Specifically, if the entire location string does not have a valid entry in its corresponding mapping dictionaries, we remove the lowest administration level from the string and repeat the mapping process. This is helpful when the lowest administration level in a string (usually city) is not present in the mapping dictionaries but the string contains information about higher administration levels that can be used to successfully identify its location (with a loss of granularity). We did not consider the Democratic Republic of Congo for two reasons. First, it was particularly difficult to differentiate DRC and Congo in self-reported free-form user locations that were used to geolocate tweets. Second, we encountered multiple variations of DRC in the user locations that made it difficult to map them appropriately using our heuristic-based geolocation algorithm.

In a random selection of 100 tweets, we found that this process correctly mapped 95% tweets. Further, the algorithm reduced false positives and improved time and space efficiency of the mapping process over the baseline.

A.3 Mapping Dictionaries

A mapping dictionary is used to look up location strings and map them to complete geographical locations. Each dictionary contains two values: *location_string* and *mapped_location*. The *location_string*, column is used to match self-reported location strings, and the *mapped_location* column is the corresponding geographical location. For instance, if the location string is "Johannesburg, South Africa", the corresponding mapping dictionary will have an entry with

medRxiv preprint doi: https://doi.org/10.1101/2025.08.19.25334033; this version posted August 24, 2025. The copyright holder for this preprint (which was not certified by peer review) is the author/funder, who has granted medRxiv a license to display the preprint in perpetuity.
It is made available under a CC-BY 4.0 International license .

location_string, as “Johannesburg, South Africa” and *mapped_location* as “Johannesburg, City of Johannesburg Metropolitan Municipality, Gauteng, South Africa”.

We create eleven such mapping dictionaries based on the number and type of administration levels (*AL*) present in a location string:

- *AL* = 1 has three dictionaries, containing either country, state, or city information in the *location_string* column.
- *AL* = 2 has six dictionaries, containing information about one of the following pairs in the *location_string* column: state-country, district-country, district-state, city-country, city-state, city-district.
- *AL* = 3 has two dictionaries, containing either distinct-state-country or city-state-country information in the *location_string* column.

A.4 Topic Modeling

We quantitatively assess the quality of topics in each set by computing coherence scores using four measures of coherence (in Table A1), U_MASS, C_V (0, 1), C_UCI (0,1), and C_NPMI (0,1) (25,26). We also reviewed the top words of topics within each topic set for a qualitative check of interpretability. Using both methods, we reached the consensus that the 200-topic model was optimal. Finally, we obtained the probability distribution for each tweet across all 200 topics.

Table A1: Comparison of coherence scores across different topic sizes using Latent Dirichlet Allocation.

**Topics**	U_{MASS}	C_V	C_{UCI}	C_{NPMI}
50	-4.936	0.621	0.561	0.094

medRxiv preprint doi: https://doi.org/10.1101/2025.08.19.25334033; this version posted August 24, 2025. The copyright holder for this preprint (which was not certified by peer review) is the author/funder, who has granted medRxiv a license to display the preprint in perpetuity.
It is made available under a CC-BY 4.0 International license .

100	-5.361	0.627	0.624	0.108
200	-5.635	0.631	0.674	0.110
